# Hybrid Raman and Partial Wave Spectroscopy Microscope for the Characterization of Molecular and Structural Alterations in Tissue

**DOI:** 10.1002/jbio.202400330

**Published:** 2024-10-27

**Authors:** Elena Kriukova, Mikhail Mazurenka, Sabrina Marcazzan, Sarah Glasl, Michael Quante, Dieter Saur, Markus Tschurtschenthaler, Gerwin J. Puppels, Dimitris Gorpas, Vasilis Ntziachristos

**Affiliations:** ^1^ Chair of Biological Imaging at the Central Institute for Translational Cancer Research (TranslaTUM), School of Medicine and Health Technical University of Munich Munich Germany; ^2^ Institute of Biological and Medical Imaging, Helmholtz Zentrum München Neuherberg Germany; ^3^ Klinik für Innere Medizin II, Universitätsklinikum Freiburg Freiburg Germany; ^4^ Division of Translational Cancer Research German Cancer Research Center (DKFZ) and German Cancer Consortium (DKTK) Heidelberg Germany; ^5^ Chair of Translational Cancer Research and Institute of Experimental Cancer Therapy, Klinikum rechts der Isar, School of Medicine and Health Technical University of Munich Munich Germany; ^6^ Center for Translational Cancer Research (TranslaTUM), School of Medicine and Health Technical University of Munich Munich Germany; ^7^ RiverD International B.V Rotterdam the Netherlands; ^8^ Munich Institute of Biomedical Engineering (MIBE), Technical University of Munich Garching b. München Germany

**Keywords:** field cancerization, light‐scattering spectroscopy, microscopy, multimodal imaging, partial wave spectroscopy, Raman spectroscopy

## Abstract

We present a hybrid Raman spectroscopy (RS) and partial wave spectroscopy (PWS) microscope for the characterization of molecular and structural tissue alterations. The PWS performance was assessed with surface roughness standards, while the Raman performance with a silicon crystal standard. We also validated the system on stomach and intestinal mouse tissues, two closely‐related tissue types, and demonstrate that the addition of PWS information improves RS data classification for these tissue types from R^2^ = 0.892 to R^2^ = 0.964 (norm of residuals 0.863 and 0.497, respectively). Then, in a proof‐of‐concept experiment, we show that the hybrid system can detect changes in intestinal tissues harvested from a tumorigenic *Villin*‐Cre, *Apc* ^fl/wt^ mouse. We discuss how the hybrid modality offers new abilities to identify the relative roles of PWS morphological features and Raman molecular fingerprints, possibly allowing for their combination to enhance the study of carcinogenesis and early cancer diagnostics in the future.

## Graphical abstract

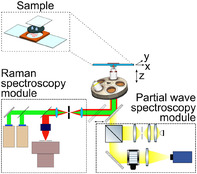

**Abbreviations**
ASM: angular second moment
BF: brightfield
CCD: charged coupled device
CV: cross‐validation
FC: field cancerization
FOV: field of view
FP: fingerprint
GG: ground glass diffusers
GI: gastrointestinal
GLCM: gray‐level co‐occurrence matrix
H&E: hematoxylin and eosin
HWVN: high wavenumber
IDM: inverse difference moment
LCTF: liquid crystal tunable filter
LEBS: low‐coherence enhanced backscattering spectroscopy
LED: light emitting diode
NA: numerical aperture
PBS: phosphate buffered saline
PCA: principal component analysis
PFA: paraformaldehyde
PLS‐DA: partial least squares discrimination analysis
PWS: partial wave spectroscopy
ROI: region of interest
RS: Raman spectroscopy
SM: spectroscopic microscopy
WT: wild type

## 1 Introduction

Field cancerization (FC), also termed field carcinogenesis, refers to widespread changes in tissue areas extending beyond the site of tumor development [1, 2]. Since these changes can precede the onset of a malignant lesion, their detection can help in risk stratification and early cancer detection [3]. Consequently, FC has been considered as the target of various advanced optical methods developed for in vivo detection [4, 5].

Morphological biomarkers, representing cellular alterations, have previously been associated with FC using technology that quantifies refractive index changes that are proportional to the amplitude and the length scale of mass density variations within a cell. Such technology includes methods such as low coherence enhanced backscattering spectroscopy (LEBS) [6, 7] and partial wave spectroscopy (PWS) [8, 9]. LEBS has been used to identify FC in various cancer types, including colorectal cancer [10], pancreatic cancer [11], and lung cancer [12], with promising results in early cancer identification. Similarly, PWS has been applied to identify colorectal, ovarian, prostate, and esophageal cancers as well as other indications [13], demonstrating that nanoscale structural alterations contain information of early cancer development. In addition to morphological changes, biochemical alterations caused by FC may also provide a distinct “fingerprint” of altered cell function that can be captured with Raman spectroscopy (RS) [14]. In oral cancer, RS has demonstrated promising results showing the identification of FC‐related effects in premalignant states and in mucosa that appears clinically normal [15, 16]. However, RS has had difficulty in distinguishing colorectal hyperplasia from healthy colon tissue in preclinical [17] and clinical [18] studies. The limitations of stand‐alone spectroscopic methods have been previously discussed [19] and have so far hampered their clinical applicability and adoption.

As opposed to optical spectroscopy methods, which offer bulk measurements, that is, a single measurement that integrates contributions from the entire region of interest (ROI) illuminated, microscopy techniques increase the measurement complexity by requiring a scanning mechanism and possibly more advanced optics, but can yield more detailed information from the sample interrogated, including spatial information and spatially resolved differences. Even though both PWS and RS have been operating as stand‐alone modalities in microscope modes, studies that can compare their performance are challenging. To date, there has been no microscope developed that combines both modes of operation.

In this work, we investigated the merits of combining morphological and molecular microscopy for FC detection by developing a hybrid Raman and PWS microscope. Since PWS requires different (lower) numerical apertures and focus over Raman, hybrid operation was achieved by a dual objective design that rotated two dedicated objectives over the same field of view (FOV). Brightfield (BF) images were also acquired with the objective employed in the PWS measurements. The PWS mode was characterized by a surface roughness standard and ground glass (GG) diffusers, whereby the Raman mode was independently characterized with a silicon crystal standard. Then, we performed two pilot studies. In the first pilot study, we employed partial least squares discriminant analysis (PLS‐DA) to interrogate whether the availability of the two modes could improve differentiation of tissue types, in particular between mouse epithelium from the stomach compared to the intestine. In the second pilot study, we interrogated whether both modes of the hybrid system could successfully identify differences in tissues obtained from control animals vs. tissues obtained from a mouse model with intestinal epithelial cell (IEC)‐specific loss of the tumor suppressor gene adenomatous polyposis coli (APC) [20]. In the latter case, the tissues were obtained from sites with no evident tumors developed. In the following, we present the methods by which our hybrid microscope was developed and our findings and discuss how such a hybrid modality could be used to research the relative role of structural and molecular signals in FC and possibly, in the future, improve diagnostics through dual‐mode operation.

## 2 Materials and Methods

### 2.1 Experimental Setup

**FIGURE 1:**
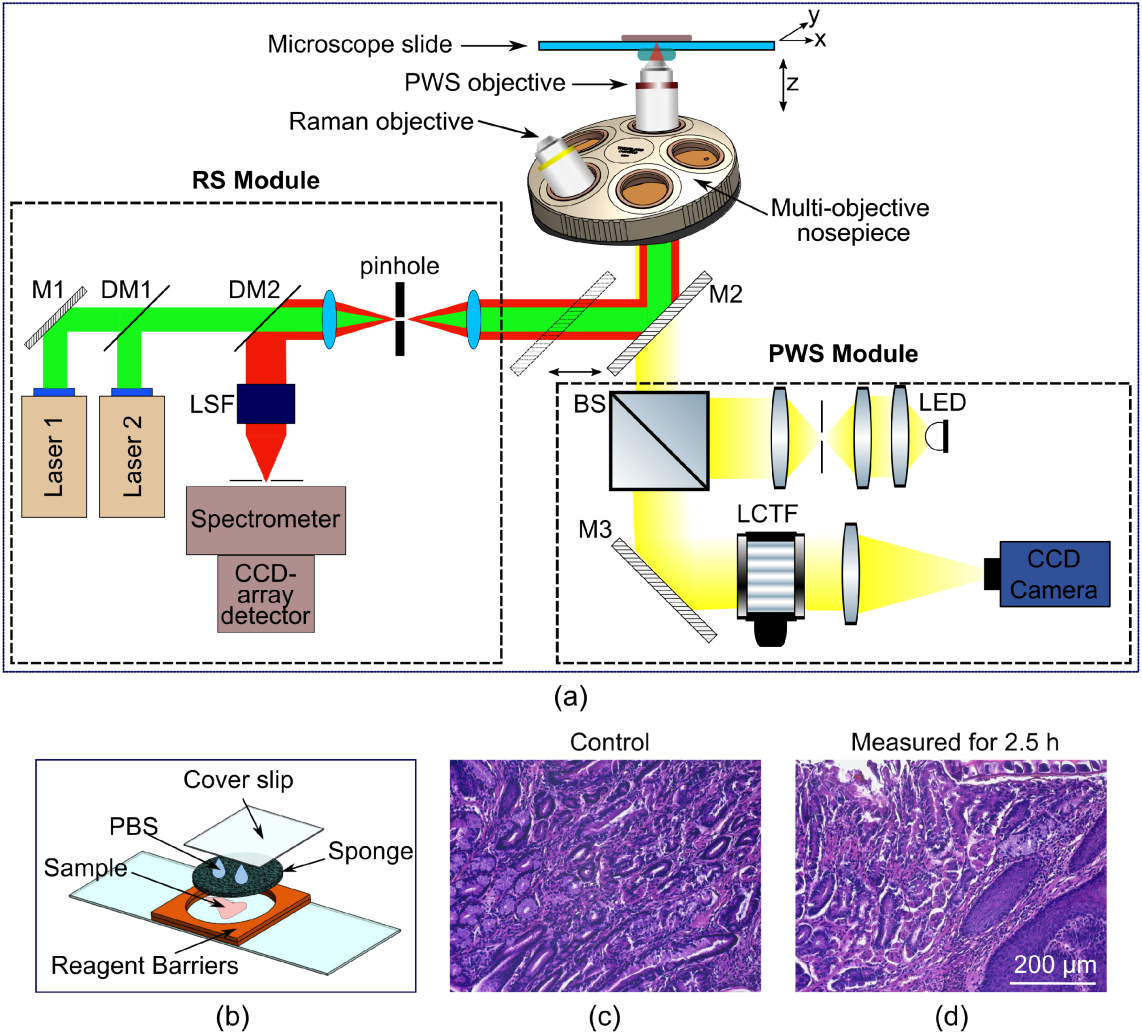
Optical setup of the multimodal hybrid Raman and partial wave spectroscopy (PWS) microscope. (a) Schematic showing the Raman spectroscopy (RS) (left dashed box) and PWS (right dashed box) modules incorporated into the microscope. BS—beam splitter, CCD—charge‐coupled device, DM—dichroic mirror, LCTF—liquid crystal tunable filter, LED—light‐emitting diode, LSF—laser suppression filter, M1 and M3—light beam turning mirrors, M2—move‐in mirror. (b) Components used to mount freshly excised thick tissue samples onto the microscope slide: PBS—phosphate buffered saline (c, d) Images of hematoxylin and eosin (H&E) stained sections of L2‐*IL1B* mouse stomach tissue (squamocolumnar junction): the control section (c) and the section that had undergone 2.5 h of measurements (d).

The hybrid Raman and PWS microscope (Figure 1a) was built with a modular design using three separate stand‐alone modules: the microscope body, the RS module, and the PWS module. The microscope body was assembled from Cerna Microscope Components (Thorlabs, Inc., Newton, NJ), and incorporates an XY stage (MLS203‐1, Thorlabs, Inc., Newton, NJ) for sample scanning and a Z stage (ZFM2030, Thorlabs, Inc., Newton, NJ) for focus adjustment. A multi‐objective nosepiece (CSN500, Thorlabs, Inc., Newton, NJ) is used to accommodate different objectives: a 1.2 NA Raman objective (RiverD International B.V., Rotterdam, the Netherlands), and a low‐numerical aperture (NA) PWS objective (RMS20X, Thorlabs, Inc., Newton, NJ). A move‐in mirror (M2) is used to switch between the two modules, RS and PWS. The microscope is built in an inverted configuration with epi‐illumination and also allows the collection of BF images through the PWS objective. The inverted configuration is well suited for imaging thin as well as thick tissue samples (> 1 mm thick).

#### 2.1.1 RS Module

The RS module was developed by RiverD (RiverD International B.V., Rotterdam, the Netherlands) and is fully optimized for efficient and reproducible Raman measurements in the red/near‐infrared part of the spectrum. The RS module is comprised of two single mode lasers (785 nm and 671 nm), a spectrometer, and a charge‐coupled device (CCD) camera. Two excitation wavelengths allow the detection of Raman signals at both the fingerprint (FP) region (~300–2500 cm^−1^) and the high wavenumber (HWVN) region (~2500–4500 cm^−1^). This choice of laser wavelengths offers an optimal balance between achieving minimum light absorption by biological samples and maximum signal detection efficiency by silicon‐based detectors. Both laser beams are collimated to a beam of 10 mm in diameter. The collimated laser beam is guided to the microscope body and focused on the tissue sample by the Raman objective, which was also developed by RiverD (RiverD International B.V., Rotterdam, the Netherlands). Backscattered light is then collected by the same objective and follows the same optical path back into the RS module. Software, also developed by RiverD, enables calibration measurements that remove Raman signatures originating from the instrument from the acquired Raman spectra, including signal from the microscope slides used for the measurements. The calibration procedure was based on five 75 × 25 mm^2^ silica microscope slides (thickness = 0.5 mm, Science Services GmbH, Munich, Germany) made from the same silica batch. Therefore, all slides share the same known Raman spectrum, allowing for system characterization and subsequent identification of the system‐specific Raman signature. The same slides were also used for all measurements of this study.

#### 2.1.2 PWS Module

The PWS imaging modality was built based on the spectroscopic microscopy (SM) system proposed by Backman [21]. Light from a broadband light emitting diode (LED) (MWWHLP1, Thorlabs, Inc., Newton, NJ) is collimated by means of a collimating lens and 1 × 1 telescope with an iris and directed into the microscope, where it is focused on the sample using the low‐NA (NA = 0.4) objective. The backscattered light is collected by the same objective, spectrally filtered by an LCTF (KURIOS‐WB1/M, Thorlabs, Inc., Newton, NJ), and imaged by a CCD camera (Grasshopper3 GS3‐U3‐28S5M, Teledyne FLIR LLC, Wilsonville, OR). A stack of 151 images is recorded for each FOV at wavelengths ranging from 550 to 700 nm with 1 nm steps. Thus, each pixel of the stack contains a tissue reflection spectrum. Before the measurements, the Instrument Response Function (IRF) was also measured by acquiring the image stack of the empty slide. The PWS image calculation was based on an approach developed by Cherkezyan et al. [22]: Firstly, a 6^th^‐order low‐pass Butterworth filter (cutoff frequency = 0.025) was applied to each spectrum. Secondly, a low‐order (*n* = 2) polynomial was fitted to the filtered spectrum and subtracted from the filtered spectrum. The subtraction corrects for the variations in the light source spectrum. The resulting spectrum was used to calculate a tissue correlation decay rate (CDR) for a particular pixel. The CDR was computed by taking the second moment of the Fourier transform of the resulting spectrum. Finally, the per pixel CDRs were combined into an image called the PWS image. Each tissue's PWS image was then divided by the IRF PWS image, calculated following the same process. In the final PWS image, each pixel was 0.463 × 0.463 μm^2^ in size. For the PWS image analysis, the region with 401 × 401 pixels in the center of the recorded images was used for all measurements.

#### 2.1.3 Determination of Long‐Term Thermal Stability of the Hybrid Raman and PWS Microscope

The hybrid Raman and PWS microscope was built inside a closed black box containing both RS and PWS modules with all associated electronics and power supplies. This design results in heat accumulation in the closed box, which could potentially result in the drift of the RS module parameters away from their calibrated values. The long‐term stability of the hybrid microscope in such conditions was assessed by recording the Raman spectra of aspirin (Bayer, Leverkusen, Germany) for both FP and HWVN regions with all the electronics in the box switched on. Twelve Raman spectra of aspirin were recorded over a period of 30 h, which included system operation for an entire workday (~7 h), being switched off and allowed to cool down overnight, and, then operation during the following workday (~7 h). It was found that laser wavelengths were stable within one pixel of the aspirin line positions for both FP and HWVN regions while the internal box temperature varied about 4.5°C during the measurement period.

### 2.2 Test Standards

**FIGURE 2:**
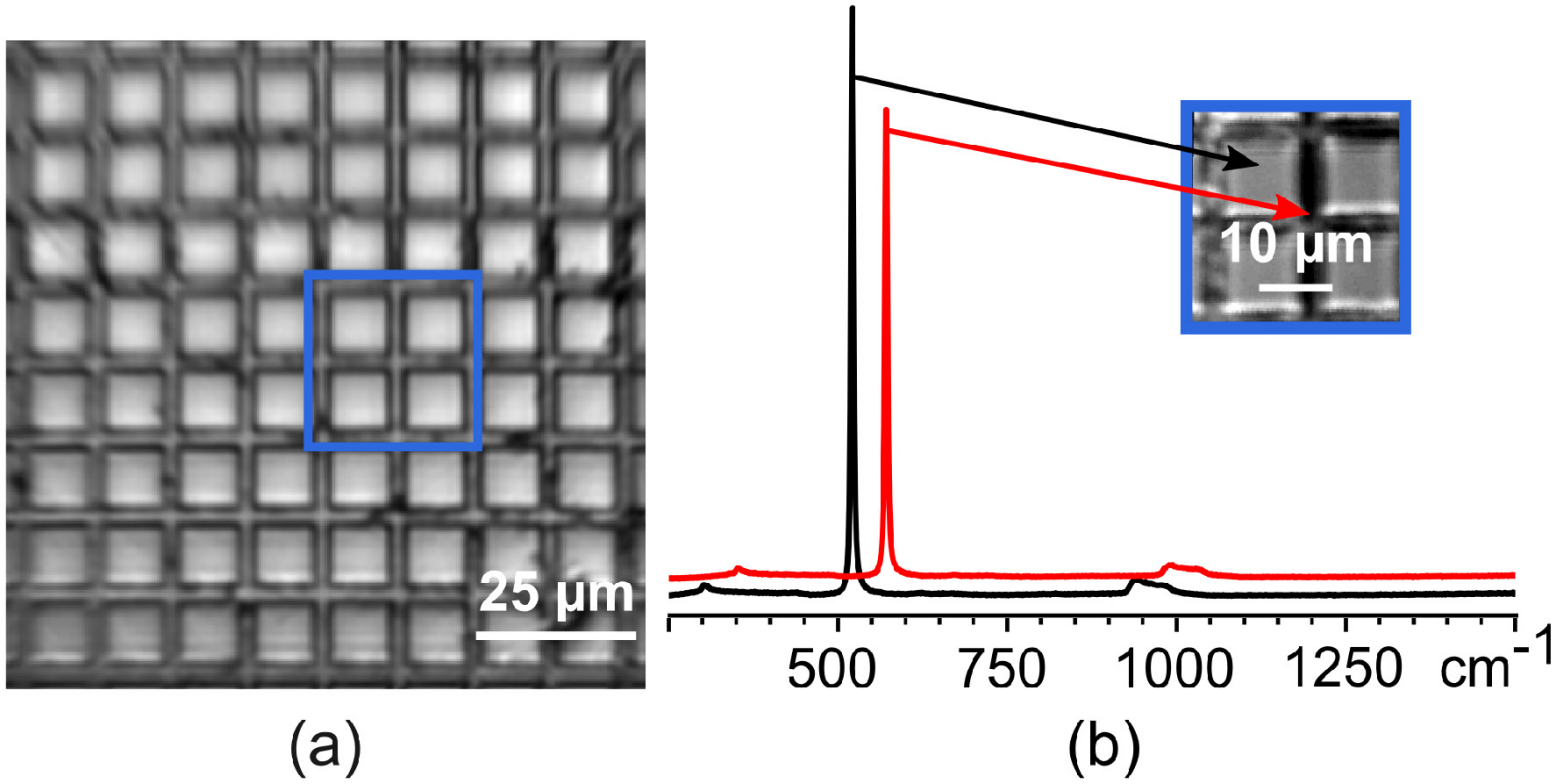
Characterization of the Raman module. (a) Brightfield image of the silicon target. The blue square marks the region of interest (ROI) for the Raman scan. (b) Raman spectra taken in and out of focus (black and red lines, respectively) from the area in outlined in blue in (a). Inset—Raman image of the selected ROI.

**FIGURE 3:**
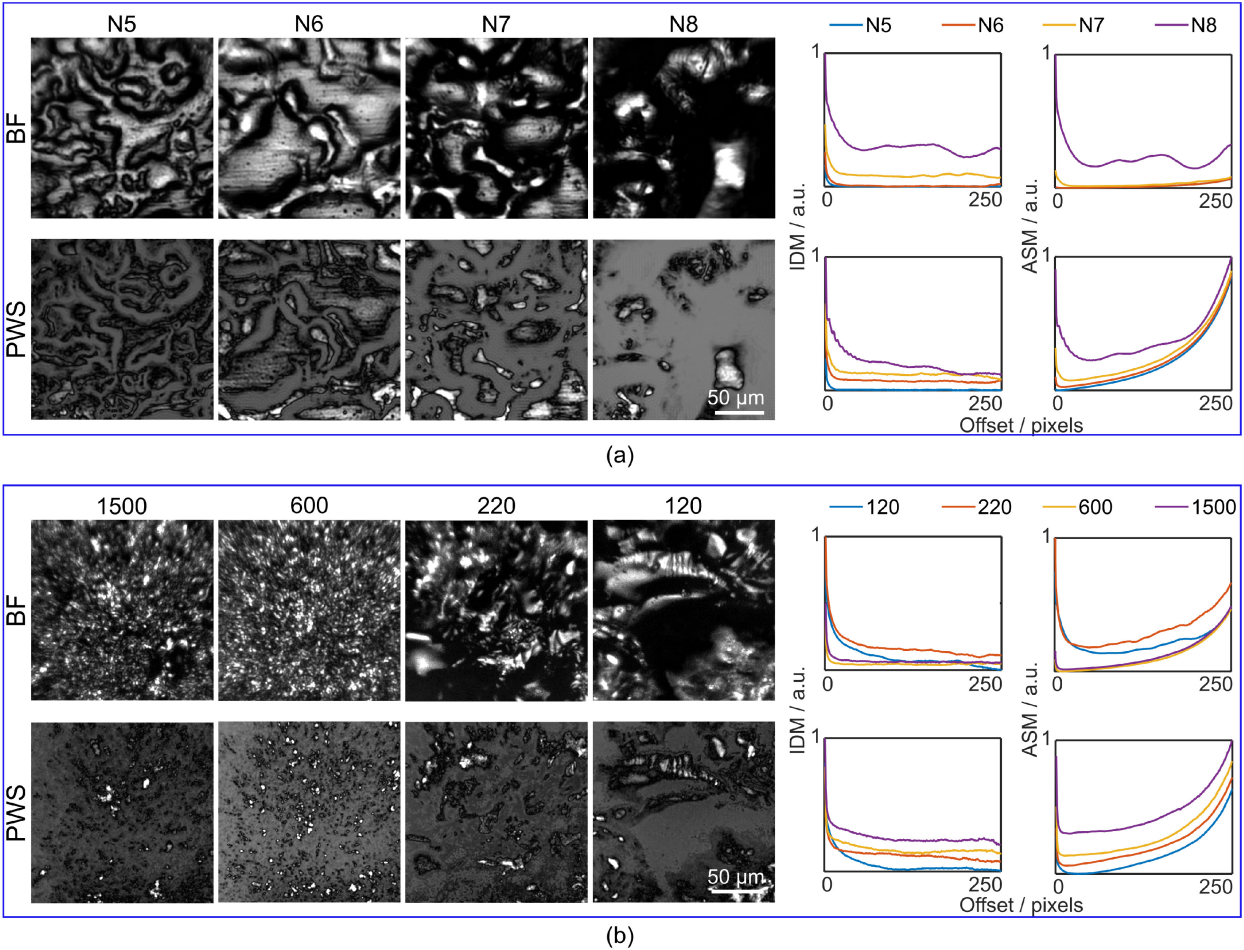
Brightfield (BF) and partial wave spectroscopy (PWS) images of the surfaces of different standards. (a) Images of reference samples (N5‐N8) from the Microsurf 331 surface roughness standard acquired with the BF and PWS modes. The textural features inverse difference moment (IDM) and angular second moment (ASM) are derived from Gray‐level co‐occurrence matrices calculated for each reference sample and are plotted on the right. (b) Images of ground glass (GG) diffusers with varying grit (1500, 600, 220, and 120) acquired with both BF and PWS; IDM and ASM plots for each grit.

A silicon test grid (Agar Scientific Ltd., Essex, United Kingdom) consisting of visible squares of 10 μm periodicity formed by electron beam lithography on single‐crystal silicon was used for the initial tests of the RS module (Figure 2). A surface roughness standard (Microsurf 331, Rubert&Co, ISO 17025:2017, Cheshire, United Kingdom) was used to assess the ability of PWS module to measure surface roughness at the micrometer scale. The standard consists of reflective rough metal surfaces. For the tests, the reference samples used were N5‐N8 with mean roughness (R_a_) values of 0.4, 0.8, 1.6, and 3.2 μm, respectively (Figure 3a). A set of GG diffusers with grit of 1500, 600, 220, and 120 (Thorlabs, Inc., Newton, NJ) was used as the second phantom for PWS microscope characterization (Figure 3b). This type of phantom mimics the diffuse reflection of biological tissue.

### 2.3 Animal Models and Histopathology

#### 2.3.1 Collection and Analysis of Mouse Stomach Tissues

One 10.5‐month‐old male L2‐*IL1B* [23, 24] mouse and ten 15‐month‐old male and female C57BL6/J wild type (WT) mice (Charles River Laboratories) were euthanized by inhalation of an overdose of isoflurane. Their stomachs were collected, opened, washed with PBS, and cut into two equal parts under a dissection microscope. One half was fixed in 4% paraformaldehyde (PFA) overnight for histopathological analysis and the other half was used for imaging. Following fixation, samples were processed for paraffin embedding and the obtained formalin‐fixed paraffin‐embedded (FFPE) sections were stained with hematoxylin and eosin (H&E).

The stomach from the L2‐*IL1B* mouse was used to test our novel measurement protocol for time‐consuming experiments, the details of which can be found in Section 2.4. For further measurements with the hybrid microscope, stomachs and intestinal (see Section 2.3.2) tissue samples from the WT mice were used.

#### 2.3.2 Collection and Analysis of Mouse Intestinal Tissues

IEC‐specific knockout mice for one *Apc* allele were generated by means of the *Villin*‐Cre/loxP system [25]. The *Apc* ^fl/wt^ allele has been previously described [20] and was crossed to *Villin*‐Cre mice [25]. Inactivation of the tumor suppressor protein APC in IECs leads to the development of widespread adenomas by 6 months of age.

One 16.5‐month‐old male *Villin*‐Cre, *Apc* ^fl/wt^ (hereafter referred to *Apc*) mouse and ten 7–18‐month‐old male and female littermate animals containing only Cre recombinase or no Cre recombinase were euthanized by isoflurane and subsequent cervical dislocation. For measurements with the multimodal system, the same parts of the small intestine were collected from both the *Apc* and control mice, opened longitudinally, and washed with PBS. In the *Apc* mouse, macroscopically normal tissue samples from sites with no evident tumors were taken for optical measurements. Adjacent sections from the same intestine which contained both tumor and normal tissue were fixed in 4% PFA and processed for H&E staining as described above. The *Apc* mouse had 12 tumors in the small intestine and no tumors in the colon.

All animal studies were conducted in compliance with European guidelines for the care and use of laboratory animals and were approved by the Institutional Animal Care and Use Committees (IACUC) of the Technische Universität München and the Regierung von Oberbayern (animal protocol number: ROB‐55.2‐2532.Vet_02‐17‐79 and ROB‐55.2‐2532.Vet_02‐15‐29). Following fixation in 4% PFA, samples for histopathological examination were embedded in paraffin. Paraffin blocks were cut into sections. The sections were stained with H&E.

### 2.4 Sample Preparation Protocol for Time‐Consuming Tissue Experiments Conducted Using the Hybrid Raman and PWS Microscope

A new protocol for mounting freshly excised tissue samples onto microscope slides was developed for measurements on the hybrid Raman and PWS microscope (Figure 1b). First, several reagent barriers (FastWells, Bio‐Labs, OR, USA) were stacked on top of each other on a 75 × 25 mm^2^ silica slide (thickness = 0.5 mm), so that the height of the stack exceeded the thickness of the tissue sample by ~1 mm. In most cases, the same number of barriers was used regardless of whether stomach or intestine tissue was attached to the slide. The tissue sample was then placed on the slide within the stacked reagent barriers, covered by a histology sponge cut to the same size as the opening of the reagent barriers. Depending on the sample type, 3 (for intestinal samples) or 4 (for stomach samples) drops of PBS were dropped on the histology sponge to keep the tissue hydrated during the measurements. Finally, a closed cell for the tissue was created by sealing the stack with an adhesive cover slip, which lightly pressed the histology sponge onto the tissue and held the tissue in place on the slide surface. During measurement, the liquid PBS created a saturated PBS vapor inside the sealed cell, preventing the sample from drying.

The new measurement protocol was tested on a fresh stomach sample from the L2‐*IL1B* mouse. The sample was cut into two parts. One part was placed on the slide as described above and measured with the hybrid microscope for 2.5 h. The second part, which was used as a control, was stored at 4°C in PBS during the measurements. Then, both parts of the sample were fixed at the same time for further histopathological examination.

### 2.5 Measurements With the Hybrid Raman and PWS Microscope

First, microscope slides with freshly excised tissue samples were placed in the slide holder of the microscope. Then, for each tissue sample, regions of interest (ROIs) were selected to ensure that measurements were done on areas containing tissue only, as explained below. The data were recorded from the same ROI for brightfield (BF), PWS and RS modes. For stomach tissue measurements, one FOV was recorded for each mouse (*n* = 10 FOVs for 10 WT mice). For intestinal tissue measurements, one to three FOVs were recorded for each mouse (*n* = 22 for 10 control mice). In the *Apc* mouse, three FOVs were recorded from intestinal tissue with no macroscopically visible tumors. Locations containing microbubbles, which occur after washing the organs with PBS, and microscopic food remnants not completely washed out during the tissue preparation stage were avoided.

PWS measurements were conducted as previously described (see Section 2.1.2). The acquisition time with the PWS modality was ~2 min for a 417 × 417 μm^2^ FOV.

After the PWS measurements, the objective was changed to an oil immersion Raman objective and the microscope was switched to the RS measurement mode. The Raman spectra were collected in a grid of 10 × 10 pixels at an interval of 50 μm steps. The exposure time for each pixel was 10 s for the FP region and 2 s for the HWVN region. The pixel size of the RS mode was 1 μm, resulting in an RS FOV that is approximately 450 × 450 μm^2^. The acquisition time per FOV with the Raman module was approximately 20–25 min. For each tissue sample, first PWS measurements were performed on all chosen FOVs, then, after the objective change, Raman measurements were done on all the same FOVs.

After each measurement and following removal of the tissue samples, rubber reagent barriers were removed from the silica slide. The slide was then carefully cleaned with a 5% solution of Detergent 8 (Sigma Aldrich, St. Louis, MO) in deionized water, first with a Q‐tip followed by sonication for 60 min in the detergent solution. After sonication, the slide was rinsed with deionized water, dried with compressed air, and stored in a plastic container. Before the next measurement, both sides of the slide were cleaned with high performance liquid chromatography grade ethanol (Sigma Aldrich, St. Louis, MO) and optics cleaning tissue using the drop and drag technique.

### 2.6 Data Preprocessing and Analysis

#### 2.6.1 Raman Spectra Preprocessing

The iModPoly method, which subtracts the fluorescence baseline using a fifth‐order polynomial [26], was used to prepare the Raman spectra for further analysis. To make spectra comparable, vector normalization was applied for each set of spectra recorded per FOV [27]. For each spectral dataset with distinct outliers, density‐based spatial clustering of applications with noise (“dbscan” function) classification was applied as an additional step to obtain the final spectral dataset (without outliers, noise or spectra from tissue types different from the targeted tissue type).

#### 2.6.2 BF and PWS Images Preprocessing

BF and PWS images of standards and tissue samples were evaluated using the gray level co‐occurrence matrix (GLCM) method [28], which has been previously applied for both the analysis of rough surfaces and the analysis of microscopic tissue images [29, 30, 31]. 12‐bit GLCMs (corresponding to the 12‐bit camera resolution) were calculated for each image using a standard function implemented in MATLAB (R2019b, Mathworks). An array of offsets from 0 to 250 pixels and four directions (horizontal, vertical, and two diagonals) was defined to calculate textural statistics from these GLCMs. The textural statistics were averaged (from four directions). Then textural features, namely the inverse difference moment (IDM) and angular second moment (ASM), were extracted from each computed GLCM. The IDM quantifies textural local homogeneity, while ASM quantifies the uniformity of the evaluated signal [31].

#### 2.6.3 Multivariate and Statistical Analysis of all Recorded Data

Principal component analysis (PCA) was utilized to reduce the dimensions of the obtained data (RS, BF, and PWS across all tissue samples). The scores of the first two components were displayed using scatter plots. 2D scatter plots enable the identification of data clusters and outliers, as well as the exploration of patterns in the dataset. For the obtained textural features, the squared Euclidean distance was computed for data points on each scatter plot to evaluate goodness of clustering in different tissue types. Based on the distance metrics, the silhouette scores (‘silhouette’ function) were also calculated. Silhouette scores allow estimates of how similar each point was to other points within the same cluster, in comparison to points in other clusters. Thus, the highest silhouette value determines, for example, which textural feature (i.e., IDM or ASM) enabled better differentiation between the groups of recorded data (i.e., different tissue types).

Partial least squares discriminant analysis (PLS‐DA) together with k‐fold (*k* = 4) cross validation (CV) was applied to differentiate between measurements from two epithelial tissues (stomach, *n* = 10; intestine, *n* = 22) and to compare the regression results for three datasets: (i) PWS data (IDM parameter values), (ii) RS data (FP region Raman spectra, averaged per FOV) and (iii) concatenated and standardized PWS and RS data (z‐scores were calculated to place datasets on the same scale for comparability). The selection of this statistical model was based on the type and amount of data [32] obtained. The model performance evaluation and the selection of the optimal number of components were conducted via k‐fold cross validation. Although the decision on the number of components used in the models tends to be subjective, in the current study, this decision was made based on a one‐sigma heuristic approach [33]. According to this approach (see details in the Supporting Information), the model for the PWS dataset had one component that covered the main variance of the dataset. On the other hand, the model used for the more dense RS dataset had two components. Finally, the model for the combined dataset required four components.

All algorithms used were implemented using MATLAB (Mathworks, Natick, MA).

## 3 Results

### 3.1 Development of the Hybrid Raman and PWS Microscope

Figure 1a depicts a schematic of our hybrid multimodal microscope, showing both the RS and PWS modules. The microscope was built in an inverted configuration using epi‐illumination. This reflection geometry enables imaging of thin as well as thick (bulk and opaque, > 1 mm) tissue samples. The illuminations of both the Raman and PWS modalities are directed through a common imaging path through two different objectives (Raman and low‐NA PWS) that are accommodated on a multi‐objective nosepiece. Backscattered signals are then recorded by different detectors for both the Raman and PWS modules (see Section 2 for details). A move‐in mirror (M2, Figure 1a) is used to switch between Raman and PWS modules. To search for the region of interest (ROI) and to record bright‐field (BF) images of these ROIs, a CCD camera (PWS module, Figure 1a) is used.

We developed a new measurement protocol in order to ensure that measurements could be reliably conducted over long periods of time without damaging the sample and tested the protocol on freshly‐excised L2‐*IL1B* mouse stomach tissue (Figure 1b–d). The sample was divided into two parts, with one part measured with the hybrid microscope for 2.5 h while the second part was set aside as a control. Following the experiment, both halves of the sample were fixed and stained for H&E. The halves were then compared to examine whether any morphological alteration had occurred in the measured sample after analysis (Figure 1c,d). The sample halves were evaluated by a trained expert (author S.M.), who determined that no significant alterations in tissue, cellular morphology, and staining intensities of the imaged sample were observed compared to the control sample. Furthermore, no tissue necrosis due to excessive tissue compression and degeneration due to dehydration was observed following imaging with the hybrid microscope. The newly established protocol was then used for further measurements of mouse stomach and intestinal tissues (Sections 3.3 and 3.4).

### 3.2 Characterizing the Hybrid Raman and PWS Microscope Using Test Standards

The Raman module was characterized using a test specimen made of single crystal silicon. The silicon test grid was placed on top of a silica slide, grid facedown with no refraction index matching liquid. Raman spectra were recorded by means of two‐dimensional raster scans with 0.1 μm steps. The reconstructed Raman image of the ROI outlined in blue in Figure 2a is shown inset in Figure 2b. The image contrast comes from the variation of the Raman intensity at 522 cm^−1^ as the specimen surface moves in and out of focus due to valleys in the grid (see Figure 2b; black and red lines, respectively). The Raman spectrum recorded with the RS module of the hybrid system corresponds to the Raman spectrum of silicon crystal taken from the RRUFF Raman database [34].

To characterize the PWS module's performance, tests were performed using two types of standards: a surface roughness standard (Microsurf 331, Rubert&Co, ISO 17025:2017) (Figure 3a) and a set of GG diffusers (Figure 3b). Images of both test standards were obtained using the PWS and BF modes and their textural features were quantified via GLCM [28] analysis (Figure 3).

Figure 3a shows BF and PWS images for the first four out of eight reference samples of the Microsurf standard, as the mean roughness (R_a_) values of these samples are within the depth of field of the PWS objective, which was calculated to be 3.44 μm. R_a_ is defined as the average deviation of a surface from the mean height. R_a_ values of the samples N5‐N8 (Figure 3a) are 0.4, 0.8, 1.6, and 3.2 μm, respectively. To extract textural features from the PWS and BF images, GLCMs were calculated for each image. The features extracted for the PWS images, ASM and IDM, show distinct separation across R_a_ values in the PWS images compared to the same features in the BF images (Figure 3a). This result implies that both textural features (ASM and IDM) decrease with the increasing roughness of the sample. Thus, PWS images allow for a more sensitive assessment of textural properties, and possibly more sensitive tissue discrimination, than BF images.

To characterize the ability of the PWS mode to measure diffuse reflection samples, a set of GG diffusers was used. GG diffusers with varying surface quality (grit: 1500, 600, 220, and 120) were imaged (Figure 3b) with both the BF and PWS modes, and the textural features ASM and IDM were extracted from the GLCM of the obtained images. Similar to the surface roughness standard test, textural properties evaluated from the PWS data (both IDM and ASM) demonstrate distinct separation between different grit sizes compared to IDM and ASM results for BF images (Figure 3b). Thus, the PWS modality can detect morphological variations on the microscale (0.4—3.4 μm) level.

### 3.3 Characterizing the Hybrid Raman‐PWS Microscope Using Freshly‐Excised Tissue Samples

**FIGURE 4:**
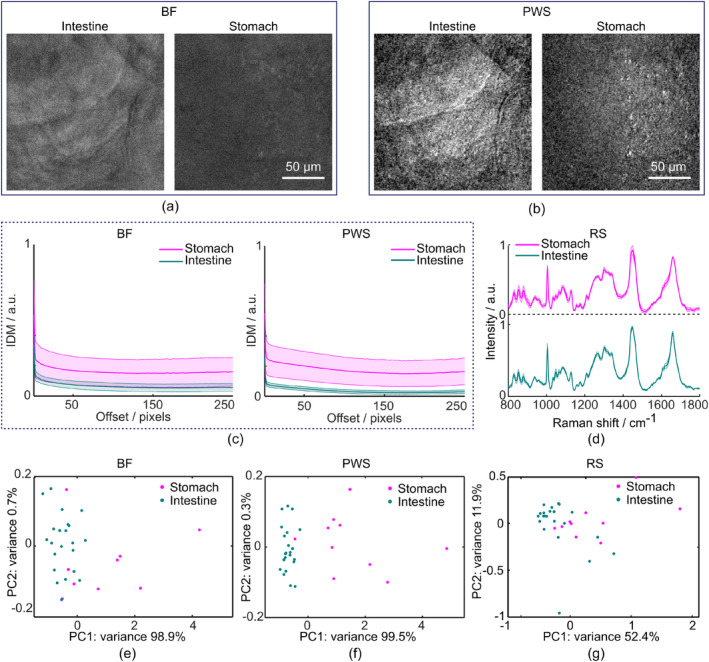
Characterization of the partial wave spectroscopy (PWS) and Raman modalities with freshly‐excised tissue samples. (a, b) Brightfield (BF) and PWS images of freshly excised samples of mouse intestine and stomach. (c) Inverse difference moment (IDM) textural feature distribution plots with means (solid line) and standard deviations (shaded sections) calculated from BF images (left) and PWS images (right) of the stomach and intestine tissue samples. (d) Raman spectra collected from the stomach and intestine samples with means and standard deviations (shaded sections) indicated; (e–g) Scatter plots resulting from principal component analysis of the data from the stomach and intestine samples for BF (e), PWS (f), and RS (g) modalities.

We then interrogated whether the availability of the two modes could improve the differentiation of closely‐related tissue types, in particular between mouse stomach and intestinal tissues. To do this, we first characterized the PWS mode on the tissue samples, using a similar method as with the measurements conducted on test standards. Figure 4a,b shows exemplary PWS images and BF images of mouse intestine and stomach samples from the same FOVs. We evaluated both textural features, IDM and ASM, however, for the tissue samples, IDM enabled better differentiation between the two groups according to the silhouette scores (0.72—for IDM, 0.55—for ASM, see Section 2 for details). IDM values for PWS and BF images of the tissue samples are shown in Figure 4c. Scatter plots of scores from the first two principal components (PCs) of the IDM measurements for both BF and PWS images are shown in Figure 4e,f. Better clustering of the tissue samples was achieved for PWS image data compared to BF image data, according to calculated silhouette values from the respective score plots (0.57 for BF, 0.72 for PWS). In addition, Raman spectra of both the stomach and intestine tissue samples were recorded from the same FOVs as the PWS and BF images for further analysis of the combined data from both Raman and PWS modes (Figure 4d).

**FIGURE 5:**
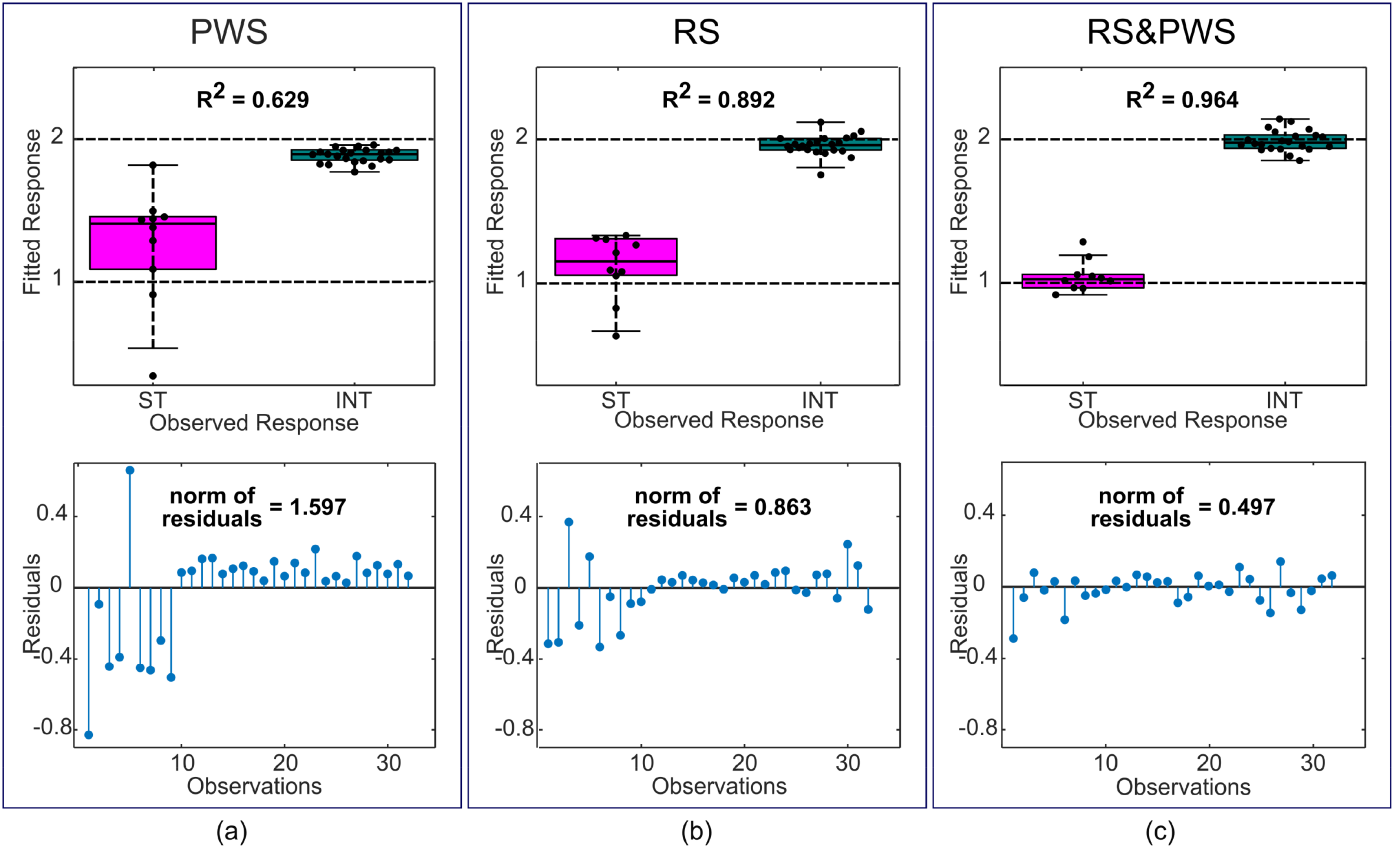
Classification results using the partial least squares discriminant analysis (PLS‐DA) algorithm for datasets obtained with each modality alone and a combined dataset. (a) Partial wave spectroscopy (PWS), (b) Raman spectroscopy (RS); (c) Merged RS and PWS. Top row: Categorical plots of the classification results with indicated R^2^ values (ST—stomach, INT—intestine). Bottom row: Residuals for each dataset with norms of residuals indicated.

We employed partial least squares discriminant analysis (PLS‐DA) to investigate whether the availability of both RS and PWS could improve classification results in mouse stomach tissue and intestinal epithelium. The results of the PLS‐DA classification model are shown in Figure 5 for two datasets (stomach FOVs, *n* = 10; intestine FOVs, *n* = 22) obtained using the PWS and RS modalities, and for the third combined dataset (RS and PWS datasets together, see Section 2). To quantify the performance of the PLS‐DA algorithm, R^2^, residual values, and the norm of residuals for every fitted model were calculated (Figure 5). From these classification results, we observe that the RS modality discriminated between stomach and intestine tissue better than PWS, with an R^2^ value of 0.892 and a norm of residuals of 0.863 compared to an R^2^ value of 0.629 and a norm of residuals of 1.597 for the PWS dataset. However, results from the combined RS and PWS datasets show improvement over results for the RS modality alone (Figure 5c). In the combined RS and PWS dataset, classification results yield the highest R^2^ value of 0.964 and the lowest norm of residuals of 0.497.

### 3.4 Proof‐of‐Concept Experiment

Following the characterization of the microscope on freshly‐excised tissue samples, we conducted a proof‐of‐concept experiment where we used the hybrid microscope to examine intestinal tissue from a genetically‐engineered mouse model for intestinal‐specific tumorigenesis (*Apc*, see Section 2.3 for details). The loss of APC function leads to the development of multifocal tumors across the whole intestine [20]. These tumors progress at varying rates, and we therefore expect to observe both molecular and morphological differences between the normal tissue sites adjacent to these visible tumors.

**FIGURE 6:**
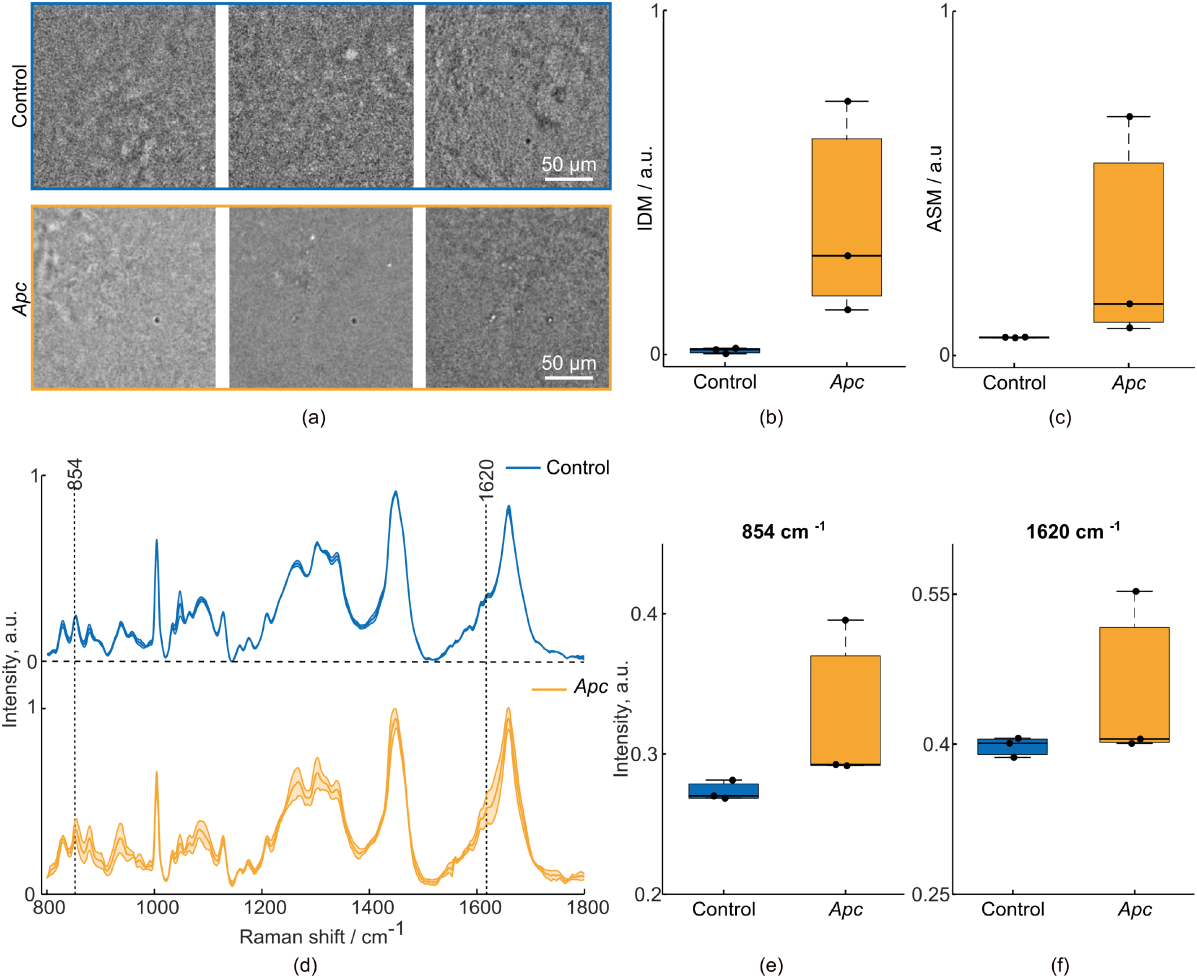
Hybrid Raman and partial wave spectroscopy (PWS) microscope measurements of intestinal samples from a control mouse (blue) and a tumorigenic *Apc* mouse (yellow). (a) PWS images of *Apc* and control intestinal samples. For each mouse, data were obtained from three fields of view (FOVs). (b) Categorical plot of the inverse difference moment (IDM) textural feature, derived from Gray‐level co‐occurrence matrices (GLCM) calculated for each PWS image. (c) Categorical plot for the angular second moment (ASM) textural feature, derived from GLCMs calculated for each PWS image. The values shown on the plots correspond to a pixel offset of 100 (see Section 2). (d) Mean Raman spectra from all intestinal tissue samples, with standard deviations indicated. Spectra are averaged per FOV. (e, f) Boxplots of intensity differences in Raman spectra between the control and *Apc* groups for Raman bands with potential diagnostic value [35, 36].

The tissue samples (*n* = 3 FOVs) were harvested from intestinal sites without macroscopically visible tumors. The results obtained from the *Apc* mouse were compared to results collected from the intestinal tissue of a control mouse. According to the tumorigenesis model, the entire intestinal tract of *Apc* mice is affected by tumor development, and thus, by FC. Therefore, we performed this test to interrogate whether both modes of the hybrid system could successfully identify molecular and structural alterations of the tissue associated with tumorigenesis and potentially FC. In Figure 6, we show results from measurements using the PWS and RS modalities from the two mice described above and compare the IDM and ASM features to Raman spectra obtained from the tissue epithelium.

Figure 6a shows PWS images of the *Apc* and control mouse tissues from each FOV. In the control group, the PWS images look very similar, which is also reflected in the IDM and ASM textural features. In contrast, PWS images from the *Apc* mouse differ both from the images of the control group and from each other, with large variations in the textural features. Figure 6b,c display the evaluated IDM (Control: 0.012 ± 0.008, *Apc*: 0.384 ± 0.315) and ASM parameters (Control: 0.053 ± 0.001, *Apc*: 0.308 ± 0.337) from GLCMs calculated from the PWS images. In agreement with the results from the previous experiment, IDM (Figure 6b) and ASM (Figure 6c) features appear to have higher values in tissues from the *Apc* mouse compared to control mouse with IDM features having a larger difference to control values. However, the ASM feature also shows differences between both groups of images (Figure 6c). Future studies on larger datasets would be required to definitively conclude which textural feature allows for better differentiation.

Figure 6d shows two groups of averaged Raman spectra per FOV. From these spectra, we observe that the standard deviation of the *Apc* group is larger than that of the control group, which is consistent with results from the PWS modality. In tissues affected by tumorigenesis, an increase is expected in Raman bands (i.e., 854 and 1620 cm^−1^) corresponding to free amino acids (e.g., tyrosine, porphyrin) [35, 36]. Boxplots (Figure 6e,f) show that the mean intensities were higher at 854 cm^−1^ (Control: 0.273 ± 0.007 vs. *Apc*: 0.327 ± 0.060) as well as at 1620 cm^−1^ (Control: 0.398 ± 0.010 vs. *Apc*: 0.453 ± 0.087) as expected, but more replicates are needed to carry out statistical tests.

## 4 Discussion and Conclusion

Advanced imaging modalities would greatly help in the diagnosis of FC by detecting, at very early stages, subtle morphological and molecular alterations that are prone to progress to cancer. Several advanced optical methods have targeted FC‐related structural or biomolecular alterations separately, as stand‐alone modalities [3, 4, 15, 16]. In the current study, we aimed to investigate the benefits of combining morphological and molecular microscopy for the detection of FC by developing a hybrid Raman and PWS microscope (Figure 1).

The PWS modality has previously only been used for the characterization of intracellular nanoscale architecture [37, 38]. To our knowledge, this study represents the first application of PWS to assess tissue structural features on the micrometer scale. During our characterization of the PWS modality, we observed that PWS images obtained from surface roughness standards allowed for a more sensitive assessment of textural properties for further discrimination purposes than the images recorded with the BF mode. This is due to the ability of the PWS modality to detect subdiffraction morphological features [39]. We also validated the PWS mode by measuring two closely related types of tissue: mouse intestine tissue and mouse stomach tissue. Both tissue types are lined with columnar epithelium, with their mucosal architecture structurally different according to the function of the respective organ [40]. Morphological differences in recorded PWS images were evaluated via GLCM, a statistical texture analysis that has been successfully applied to allow different types of tissues to be distinguished through subtle changes in light scattering [29, 30, 31]. GG diffusers (Figure 3b) display higher IDM for surfaces made up of smaller particles (i.e., those with higher grit). Our results from the GLCM texture analysis of both tissue types suggest that stomach samples exhibit higher IDM textural features values compared to intestinal samples (Figure 4d). Thus, tissues with higher IDM values (stomach tissues), could consist of smaller structures compared to tissues with lower IDM values (intestinal tissues). Such a finding would be in accordance with data obtained from scanning electron microscopy (SEM) [40] and laser‐scanning confocal microscopy [41] experiments. Furthermore, by means of PLS‐DA, we demonstrate that the structural information obtained by the PWS modality combined with molecular information obtained by RS yields the best data classification results for two types of closely‐related epithelial tissues (stomach and intestine). In addition, pilot results from both modalities demonstrate the ability to detect differences between macroscopically normal tissue from an intestinal tumorigenesis mouse model (*Apc*) and healthy tissue from a control mouse. A future study involving a larger set of animals has already been planned in order to statistically confirm the differences in spectral and textural features that are observed in Figure 6.

Therefore, we demonstrate that our hybrid RS‐PWS system can provide information from both modes, meaning that both biochemical and structural properties of tissues can be examined at the same time. Moreover, since the reflectance mode of Raman modality has already been exploited for in vivo endoscopic use, in vivo endoscopic human measurements using a system with combined RS‐PWS modalities is potentially possible.

Several other multi‐modal approaches have been developed, including two‐photon excitation‐fluorescence (2PEF), three‐photon excitation‐fluorescence (3PEF), second‐harmonic generation (SHG), third‐harmonic generation (THG), coherent anti‐Stokes Raman scattering (CARS), optical coherence tomography (OCT) and fluorescence lifetime imaging microscopy of endogenous fluorophores (FLIM) [42, 43, 44, 45, 46, 47]. However, the majority of the other multi‐modal approaches are based on nonlinear optical effects and target specific biomarkers, limiting the diagnostic value of the systems. For example, multiphoton fluorescence (2PEF and 3PEF) targets mainly particular endogenous fluorophores, while multi‐harmonic generation modes (SHG, THG) are sensitive to fiber structures, like myosin and collagen, and CARS is used to detect lipid vibrational modes. On the other hand, RS can simultaneously capture changes in a variety of biomarkers, as shown here, making it an ideal candidate for early disease diagnosis. Its combination with PWS adds the morphological aspect that is frequently missing from other modalities. In the future, we believe that our system can also be expanded by the addition of complementary nonlinear label‐free modalities, like 2PEF, SHG, FLIM, and/or THG.

This study describes a novel multimodal system that integrates PWS and RS techniques, utilizing an inverted configuration with epi‐illumination to image the same FOV with both modalities. The results from these experiments demonstrate that the presented system allows for synchronous evaluation of the biochemical and structural properties of tissue. This hybrid microscope could be used to explore the relative role of structural and molecular alterations in FC and possibly, to enhance the study of carcinogenesis and early cancer diagnostics in the future through dual‐mode operation.

## Author Contributions

M.M., G.J.P., and D.G. conceptualized and designed the multimodal RS and PWS microscope. M.M. developed and characterized the microscope and collected all data. G.J.P. developed the Raman module. M.M. and S.M. designed and validated the fresh tissue measurements protocol. S.M., S.G., and M.T. handled the animal models, collected the tissue samples, and prepared them for imaging. E.K. developed the algorithms and performed the analysis of PWS, RS, and combined data. E.K., M.M., M.T., D.G., M.Q., D.S., and V.N. interpreted the outcomes of the study. E.K. wrote, reviewed, and edited the paper. D.G., G.J.P., and V.N. conceptualized the study and secured funding. All authors contributed to the writing and review of the manuscript.

## Conflicts of Interest

V.N. is a founder and equity owner of Maurus OY, sThesis GmbH, iThera Medical GmbH, Spear UG, and I3 Inc. G.J.P. is the managing director and shareholder of RiverD International BV.

## Supporting information


Data S1.

## Data Availability

The data that support the findings of this study are available from the corresponding author upon reasonable request.
