# Supplementary material for: Hybrid Raman and Partial Wave Spectroscopy Microscope for the Characterization of Molecular and Structural Alterations in Tissue
Source: J Biophotonics. 2024 Oct 27;17(12):e202400330. doi: 10.1002/jbio.202400330 (PMC11614561; doi:10.1002/jbio.202400330)
Supplement: Supplementary file 1 — Data S1. [file JBIO-17-e202400330-s001.docx]

Supporting information for **"**Hybrid Raman and partial wave spectroscopy microscope for the characterization of molecular and structural alterations in tissue”.

**Manuscript ID**: jbio.202400330

First, the components accounting for >99% of the variance (see Figure S1 a-c) was input into the PLS-DA algorithm to estimate the mean squared error of prediction (MSEP) by 4-fold cross-validation. Second, within one standard error from the global minimum MSEP value, the minimum number of components was used when running the final model for each dataset. The global minima of the MSEP curves are indicated with vertical dotted lines on the MSEP plots (Figure S1 d-f), and the horizontal dashed lines on the MSEP plots show the threshold value upon which the suggested ‘Optimal number of components’ is based. Therefore, the number of components for each model is the minimum number under the dashed line on the plots shown in supplementary Figure S1 d-f.


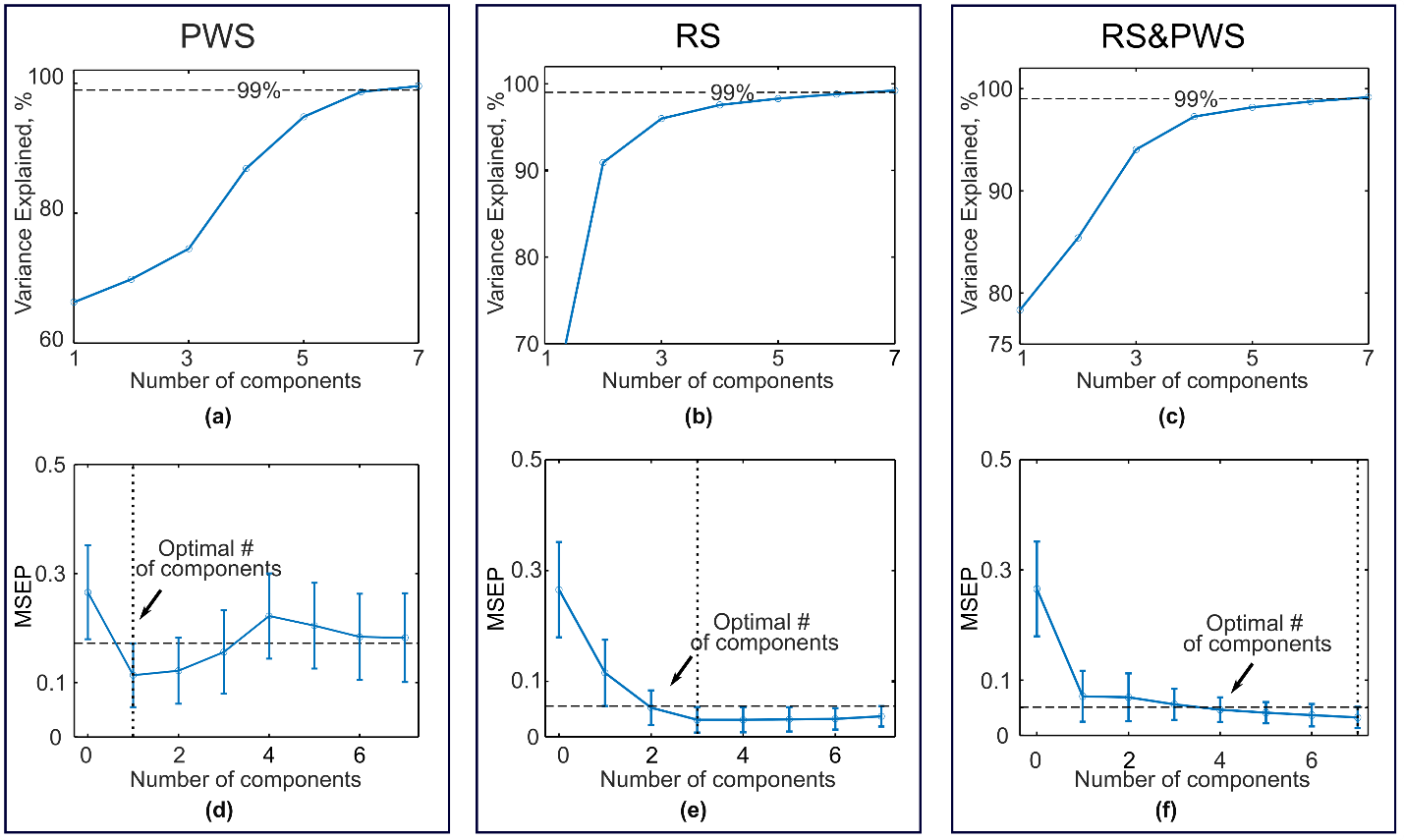


**Figure S1**. **The mean squared prediction error (MSEP) and explained variance of the partial least squares discriminant analysis (PLS-DA) models for each dataset:** **a,d)** Partial wave spectroscopy (PWS); **b,e)** Raman spectroscopy (RS); **c,f)** RS&PWS. The optimal number of components corresponds to the minimum number under the dashed line in panels d-e.
